# Trial in progress: phase I study of non-viral gene-modified CAR-T cell therapy for malignant solid tumors expressing EPHB4 receptor (CARTiEr)

**DOI:** 10.3389/fonc.2025.1633324

**Published:** 2025-08-06

**Authors:** Chikako Funasaka, Yoichi Naito, Hitomi Kubota, Yukiko Ishiguro, Nozomu Fuse, Masashi Wakabayashi, Akihiro Sato, Junichiro Yuda, Genichiro Ishii, Toshihiro Suzuki, Kazumasa Takenouchi, Tetsuya Nakatsura, Konomi Morita, Yoichi Inada, Miyuki Tanaka, Yozo Nakazawa, Shigeki Yagyu, Toshihiko Doi

**Affiliations:** ^1^ Department of Experimental Therapeutics, National Cancer Center Hospital East, Kashiwa, Chiba, Japan; ^2^ Department of Medical Oncology, National Cancer Center Hospital East, Kashiwa, Chiba, Japan; ^3^ Department of General Internal Medicine, National Cancer Center Hospital East, Kashiwa, Chiba, Japan; ^4^ Clinical Research Support Office, National Cancer Center Hospital East, Kashiwa, Chiba, Japan; ^5^ Department of Hematology, National Cancer Center Hospital East, Kashiwa, Chiba, Japan; ^6^ Department of Pathology and Clinical Laboratories, National Cancer Center Hospital East, Kashiwa, Chiba, Japan; ^7^ Division of Cancer Immunotherapy, Exploratory Oncology Research and Clinical Trial Center, Kashiwa, Chiba, Japan; ^8^ A-SEEDS Co., Ltd., Matsumoto, Nagano, Japan; ^9^ Department of Pediatrics, Shinshu University School of Medicine, Matsumoto, Nagano, Japan; ^10^ Center for Advanced Research of Gene and Cell Therapy (CARS), Shinshu University School of Medicine, Innovative Research & Liaison Organization, Matsumoto, Nagano, Japan

**Keywords:** ewing sarcoma, solid tumor, EphB4, CAR-T cell therapy, piggyBac transposon

## Abstract

**Background:**

Ephrin type-B receptor 4 (EPHB4) is overexpressed on the surface of various tumor cells, including cells from malignant bone and soft-tissue tumors. AP8901 CAR-T cell therapy can specifically recognize and kill EPHB4 receptor-expressing malignant tumor cells by modifying the natural EPHB4 receptor ligand, ephrin B2. AP8901 is being developed via genetic manipulation involving the “piggyBac transposon” and “genetically modified feeder cell” methods, which enables the stable expression of CAR proteins in T cells and prevents T cell exhaustion. AP8901 has demonstrated therapeutic efficacy and tolerability in mice transplanted with rhabdomyosarcoma cells. We planned a phase I study to evaluate the safety and efficacy of AP8901 for metastatic solid tumors.

**Methods:**

This is a single-center, single-arm, dose-escalation, phase I study to evaluate the safety, tolerability, pharmacokinetics/pharmacodynamics, and preliminary anti-tumor activity of a single intravenous dose of AP8901 in patients with Ewing sarcoma or solid tumors expressing the EPHB4 receptor. Key inclusion criteria include the following: subjects with histologically diagnosed Ewing sarcoma or solid tumor with confirmed metastasis or recurrence/no standard treatment for metastasis or recurrence, or refractory or intolerant to standard treatment; measurable or evaluable disease according to the Response Evaluation Criteria in Solid Tumors (RECIST) version 1.1; recent biopsy or surgical resection specimens with prescreening immunohistochemistry positive for EPHB4 in ≥1% of tumor cells; ECOG performance status 0 or 1; and subjects expected to survive ≥3 months from the date of enrollment. This study is being conducted at the National Cancer Center Hospital East, Japan.

**Discussion:**

The advantage of AP8901 is that it is expected to prevent T cell exhaustion and maintain its anti-tumor effect. This phase 1 study of AP8901 will provide new evidence for the application of this novel CAR-T cell therapy in patients with solid tumors, including Ewing sarcoma.

## 1 Introduction

Malignant neoplasms are the leading cause of death worldwide, with 19,976,499 new cases of cancer diagnosed and 9,743,832 deaths in 2022 (1). Approximately 21.8% of men and 18.5% of women are considered at risk of developing cancer before the age of 75 years. Cancer-related morbidity and mortality rates are both increasing as the population ages. Although recent new chemotherapies, molecularly targeted drugs, and immune checkpoint inhibitors have improved treatment outcomes, improvements have been limited to certain cancers, excluding rare cancers such as sarcomas. New therapies are therefore needed to improve the outcomes of these malignancies.

Malignant bone and soft-tissue tumors are among the rarest types of cancer. Bone and soft-tissue sarcomas tend to occur more frequently in children and young adults but are rare in older individuals, accounting for only 3% of all tumors in adults. According to estimates from cancer statistics in Japan, the incidence of osteosarcoma is 0.59 per 100,000 population and that of soft-tissue tumors is 3.60 per 100,000 population (1). Few standard treatments are available for these cancers. Small round cell sarcomas such as Ewing sarcoma and rhabdomyosarcoma are highly responsive to chemotherapy and radiation therapy, and multidisciplinary treatment combining surgery and these adjuvant therapies has been established (2). However, for advanced cases of refractory small round cell sarcomas that have relapsed after treatment, the response rate to chemotherapy is low (2). Multiple systemic chemotherapy regimens are used for the treatment of relapsed Ewing sarcoma, such as cyclophosphamide/topotecan, gemcitabine/docetaxel, high dose ifosfamide, and temozolomide/irinotecan (3). However, the response to systemic chemotherapy is approximately 30%–40% (3) and the survival of patients with relapsed Ewing sarcoma is approximately 10%–20% (4). Thus, a highly effective treatment for this patient population is needed.

Ephrin type-B receptor 4 (EPHB4) is a 185 kDa receptor-type tyrosine kinase encoded by the EPHB4 gene located on chromosome 7 (5). EPHB4 and its natural ligand ephrin B are expressed on the plasma membrane and are involved in regulating cell adhesion and cell motility during embryonic angiogenesis (6). Expression of these genes is low or almost absent in mature tissues, except in some organs (7). Thus, they may be a potential target for anticancer therapy. EPHB4 protein was shown to be overexpressed on the surface of tumor cells in malignant bone and soft-tissue tumors and in breast, ovarian, lung, prostate, and colorectal cancers (**Table 1**). EPHB4 is believed to be actively involved in promoting cancer cell growth and deterioration in these types of cancers. EPHB4 expression in malignant bone and soft-tissue tumors, particularly rhabdomyosarcoma, has been suggested to be associated with prognosis (8–10). Notably, EPHB4 was found to be highly expressed in many malignant solid tumors in adolescents and young adults, including malignant bone and soft-tissue tumors, and breast and ovarian cancers (**Table 1**).

**Table 1:** Expression of EPHB-4 in several cancers.

Cancer types	Expression (%)
Rhabdomyosarcoma (8–10)	Embryo type 5 %, 93.5 %
Lung cancer (11)	14 %
Breast Cancer (12, 13)	58 %-66.9 %
Colorectal cancer (14)	39 %
Esophagus cancer (15)	33 %
Uterus Cancer (16)	100 %
Ovarian Cancer (17)	86 %
Prostate Cancer (18)	66 %
Urothelial Cancer (19)	94 %
Mesothelioma (20)	Epitheloid 85 %, sarcomatoid 38 %

Chimeric antigen receptor (CAR) is an engineered chimeric protein combining a single-chain antibody that binds to cell surface antigens of cancer cells with intracellular signaling regions such as CD28, 4-1BB, and CD3 chains, inducing T cell activation. CAR-T cell cancer therapy kills cancer cells via a different mechanism to conventional anticancer drugs or radiation therapy and is expected to be an effective treatment for refractory or resistant cancers. The efficacy of CAR-T cell therapy has already been demonstrated in hematopoietic tumors, such as acute lymphoblastic leukemia and malignant lymphoma (21, 22). While CAR-T cell therapy is also being investigated in relation to solid tumors, its reported therapeutic efficacy is lower than in hematopoietic tumors (23), possibly as a result of immune exhaustion of CAR-T cells.

**Figure 1:**
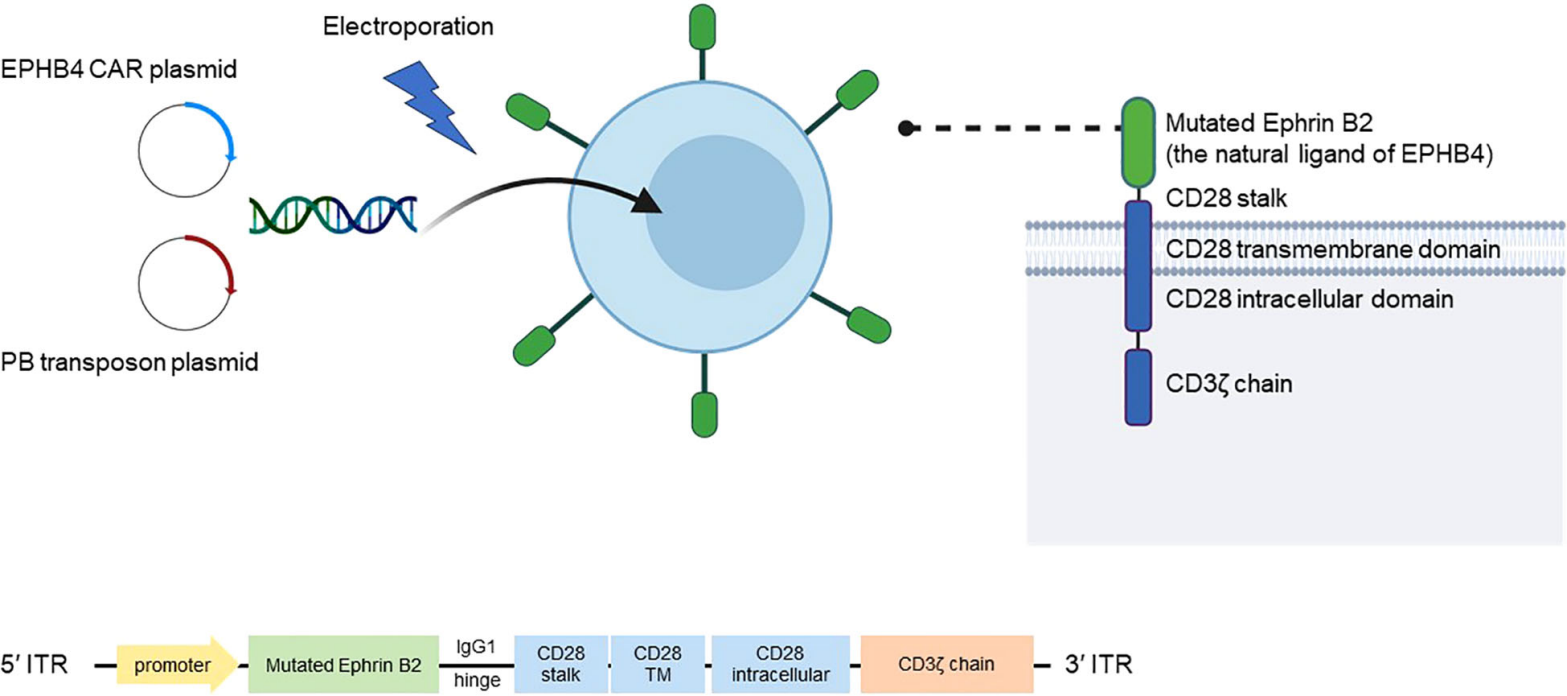
Mechanism of AP8901. AP8901 is a CAR-T cell product that targets EPHB4 via a CAR construct incorporating a mutated form of ephrin B2, the natural ligand of the EPHB4 receptor. The CAR molecule includes an IgG1 hinge, CD28 stalk, CD28 transmembrane and intracellular domains, and the CD3ζ signaling domain. The CAR transgene is delivered into T cells via electroporation using a PB transposon system, which includes a CAR-encoding transposon plasmid and a PB transposase plasmid. Stable integration of the CAR construct between ITRs enables sustained expression in T cells. The product is manufactured using both the PB-based gene transfer and a genetically engineered feeder cell expansion system. CAR, chimeric antigen receptor; EPHB4, Ephrin type-B receptor 4; ITRs, inverted terminal repeats; PB, piggyBac; TM, transmembrane.

Yagyu et al. have developed a technology to produce a variety of CAR-T cells using a genetic manipulation technique involving the “piggyBac transposon” and “genetically engineered feeder cell” methods (23). The piggyBac transposon method uses piggyBac transferase to achieve stable expression of the CAR protein in T cells (24). The researchers developed an EPHB4-CAR-T cell (product name AP8901) that can specifically recognize and kill malignant tumor cells expressing the EPHB4 receptor by modifying ephrin B2, the natural ligand of the EPHB4 receptor (**Figure 1**). The authors developed and patented a new CAR-T cell production method using a “non-viral gene modification” method that is distinct from methods for conventional CAR-T cells; this approach is cheaper, simpler, and free from possible contamination by replication-competent viral vectors. By combining these technologies, it is possible to produce EPHB4-CAR-T cells that are rich in memory T cells, which define the anti-tumor effect of CAR-T cells, but which are less immune-depleting and more effective. Transgenic CAR includes the modified natural ligand of EPHB4, ephrin B2, which specifically recognizes human EPHB4, a human IgG1 hinge region, human CD28 (transmembrane domain and part of the intracellular domain), and human CD3ζ (part of the intracellular domain) (**Figure 1**) (25). When this CAR recognizes EPHB4-expressing cells, it induces transduced T cells to acquire effector functions such as activation, proliferation, and cytotoxicity, which may in turn be effective in killing EPHB4-positive tumor cells.

In a preclinical study, Yagyu et al. used the piggyBac transposon method to produce EBHB4-CAR-T cells with a CAR-positive rate of 77.7% in 14 days (24). The resulting EBHB4-CAR-T cells had a higher CD4/CD8 ratio (0.214) and higher CD45RA+/CCR7+ positivity (62.9%), a known stem cell memory phenotype, compared with control T cells (24). EPHB4-CAR-T cells also demonstrated potent and durable efficacy against EPHB4-positive RH30 rhabdomyosarcoma cells (25). A study investigating the binding and killing potency of human EPHB4-CAR-T cells against cynomolgus monkey EPHB4-expressing cells showed that the EPHB4 CAR protein had similar binding potencies to human and cynomolgus monkey EPHB4, and human EPHB4-CAR-T cells recognized and reduced the number of cynomolgus EPHB4-expressing cells, indicating that cynomolgus monkeys could be a potential model for evaluating on-target/off-tumor toxicity (26). Indeed, toxicity evaluation in cynomolgus monkeys showed no differences in general condition, body weight, or food consumption between the test and control groups during the observation period and no differences between the two groups in terms of blood biochemistry, coagulation function tests, or pathology at necropsy (26).

These results suggest that AP8901 therapy may become a new therapeutic option, with high efficacy, in patients with EPHB4-positive solid tumors for whom there is currently no established standard of care, especially solid tumors such as Ewing sarcoma and other cancers with high EPHB4 expression detected by immunohistochemistry. We are therefore conducting a first-in-human phase I study to evaluate the safety and efficacy of AP8901 in patients with metastatic solid tumors.

## 2 Materials and analysis

### 2.1 Study setting

This is an open-label, single-arm, single-center phase I study to evaluate the safety, tolerability, efficacy, and pharmacokinetic profile of a single intravenous dose of AP8901 in patients with metastatic or recurrent Ewing sarcoma or solid tumors expressing the EPHB4 receptor.

#### 2.1.2 Study eligibility

##### 2.1.2.1 Inclusion criteria

The inclusion criteria are as follows:

1. Patients who provide free and voluntary written consent to participate in the clinical trial;
2. Age ≥18 years on the date consent is obtained;
3. Histological diagnosis of Ewing sarcoma or solid tumor with confirmed metastasis or recurrence;
4. No standard treatment for metastasis or recurrence;
5. Measurable or evaluable disease according to the Response Evaluation Criteria in Solid Tumors (RECIST) guidelines, version 1.1;
6. Most recent biopsy or surgical resection specimen with ≥1% EPHB4-positive tumor cells by immunohistochemistry with prescreening tests;
7. ECOG Performance Status 0 or 1;
8. Expected survival ≥3 months from the date of enrollment;
9. Adequate organ function measured by the following laboratory tests taken within 7 days prior to enrollment (same day of the week acceptable):
  - Neutrophil count ≥1,000/mm^3^;
  - Platelet count ≥100,000/mm^3^;
  - Hemoglobin ≥8.0 g/dL;
  - Serum creatinine **≤**2.0 mg/dL or calculated creatinine clearance (Cockcroft–Gault formula) or measured value ≥50 mL/min (Cockcroft–Gault formula: creatinine clearance value = (140 – age) × weight (kg)**/**(72 × serum creatinine value) (for women, further multiply the obtained value by 0.85);
  - Total bilirubin **≤**2.0 mg/dL;
  - Alanine aminotransferase and aspartate aminotransferase **≤**150 U/L;
  - Prothrombin time **≤**1.5 × upper limit of normal (ULN);
  - Activated partial thromboplastin time **≤**1.5 × ULN;
10. Not pregnant. Women with childbearing potential require a negative pregnancy test within 7 days prior to enrollment (same day of the week is acceptable);
11. Men and women with childbearing potential agree to use contraception from the time of consent until ≥12 months after AP8901 administration;
12. Patients willing to participate in a long-term observational study.

##### 2.1.2.2 Exclusion criteria

The exclusion criteria are as follows:

1. Patients with a current overlapping cancer or history of overlapping cancer within 2 years;
2. History of active overlapping cancer, except for intraepithelial cancers such as basal cell or squamous cell carcinoma with appropriate treatment, cervical intraepithelial carcinoma, non-invasive breast cancer, and gastric intramucosal carcinoma with complete resection. Iatrogenic ipsilateral or bilateral breast cancer not considered as a double cancer if curative treatment of one cancer has been completed and the patient has been disease-free for ≥5 years;
3. Brain metastases or spinal cord compression syndrome, unless the patient is asymptomatic and does not require treatment;
4. Breast-feeding, even if breast-feeding has been interrupted;
5. Patients receiving systemic corticosteroids or systemic immunosuppressive drugs; however, prednisolone equivalent **≤**10 mg/day is acceptable;
6. Patients receiving antineoplastic agents (e.g., chemotherapy, molecular targeted therapy, antibody therapy, hormonal therapy, endocrine therapy, and immunotherapy), other unapproved drugs, surgical therapy, radiotherapy, and radiopharmaceuticals within 14 days prior to registration;
7. Patients treated with live vaccines within 28 days prior to enrollment;
8. Confirmed HIV infection;
9. Confirmed adult T cell leukemia virus infection;
10. Active hepatitis B (HB), diagnosed as follows:
  - HBs antigen positive;
  - HBs antibody or HBc antibody positive; however, patients may be enrolled if HBV-DNA is **<**20 IU/mL (1.3 logIU/mL);
11. Confirmed active hepatitis C virus infection;
12. Active bacterial or viral infection or suspected of having these infections;
13. History of at least one treatment with any gene therapy (mRNA-based and DNA-based vaccines are acceptable);
14. History of allogeneic hematopoietic stem cell transplantation;
15. Active autoimmune disease.

### 2.2 Treatment methods

AP8901 will be administered as a single intravenous dose. AP8901 dosing will be conducted in dose-escalation studies with three to six participants at each level for the following three doses (total n=6–18), starting with dose level 1.

Doses are calculated based on body weight at the time of peripheral blood mononuclear cell collection. Autologous peripheral blood mononuclear cells will be collected from enrolled patients by apheresis for AP8901 production, after eligibility determination. If the first peripheral blood mononuclear cell collection does not provide the required cell volume for each dose level, these cells will not be used, and a second peripheral blood mononuclear cell collection will be performed. Patient-derived mononuclear cells will be filled into cell transport bags following the protocol and transported to the manufacturing facility at a controlled temperature of 2–8°C. The CAR-T positivity rate (%) of CAR-expressing cells will be set at 5%, and the shipment decision will be made after testing and characterization. After being frozen in the program freezer following the procedure manual, the samples will be stored in the vapor phase (below −150°C) of a liquid nitrogen tank and transported to the investigational sites. AP8901 will be produced using peripheral blood mononuclear cells collected from the respective participants.

Enrolled patients will receive cyclophosphamide and fludarabine the day before AP8901 administration (Day −8 to Day −1) at the following doses and schedules: fludarabine (25 mg/m^2^ intravenous once daily for 3 days) and cyclophosphamide (250 mg/m^2^ intravenous once daily for 3 days, starting at the time of the first dose of fludarabine). The following alternative regimen may be used in patients with a history of cyclophosphamide-induced grade 4 hemorrhagic cystitis or patients resistant to the most recently administered cyclophosphamide-containing regimen of lymphocyte-removal chemotherapy: cytarabine (500 mg/m^2^ IV once daily for 2 days) and etoposide (150 mg/m^2^ IV once daily for 3 days, starting at the time of the first dose of cytarabine).

An intravenous infusion of AP8901 will be administered after completion of lymphocyte-removal therapy.

### 2.3 Endpoints

The primary endpoint is the incidence of dose-limiting toxicities (DLTs) from the start of lymphocyte-depletion therapy until 28 days after treatment with AP8901. DLTs are defined as adverse events during the DLT assessment period for which a causal relationship with AP8901 cannot be excluded. Adverse events are assessed using the Common Terminology Criteria for Adverse Events (CTCAE) version 5.1 (**Table 2**). Adverse events considered to be due to disease progression are excluded from the definition of DLT.

**Table 2:** Definition of DLT.

Definition of DLT	
Hematological toxicity	• Grade 4 or Grade 3 thrombocytopenia with bleeding • Grade 4 neutropenia lasting more than 5 days • Febrile neutropenia of Grade 3 or above • Red blood cell/platelet transfusion required If transfusion of red blood cells or platelets is required.
Non-hematological toxicity	Grade 3 or higher non-hematologic toxicity except for the following non-hematologic toxicities; • Grade 3 CRS that can be controlled by anticytokine therapy • Grade 3 gastrointestinal symptoms that can be controlled by supportive care • Grade 3 or higher laboratory abnormalities that are considered clinically unproblematic • Grade 3 hypertension that can be controlled by pharmacological therapy Failure to administer AP8901 as prescribed due to Grade 3 or higher non-hematologic toxicity for which a causal relationship to lymphocyte-depleting chemotherapy cannot be denied
General	Any other toxicity (regardless of severity) deemed by the investigator and the study coordinating committee to be of any severity that is deemed equivalent to a DLT

DLT, dose-limiting toxicity; CRS, cytokine release syndrome.

Secondary endpoints are adverse events assessing using CTCAE, objective response rate (ORR), disease control rate (DCR), progression-free survival (PFS), overall survival (OS), duration of response (DoR), and pharmacokinetics (CAR gene copy number measurements).

We will also conduct the following exploratory analyses: determination of the number of CAR-positive cells in peripheral blood, phenotypic analysis of CAR-T cells, T cell receptor analysis in CAR-T cells, single-cell RNA sequencing, cytokine testing, plasma protein assay, functional analysis of CAR-T cells using AP8901 residues and residual samples from the AP8901 manufacturing process, and CAR gene expression analysis and immunostaining using secondary cancer samples.

### 2.4 Follow-up

The assessment period for adverse events and treatment failure is defined as the time from the date of peripheral blood mononuclear cell collection to 1 year after the date of administration of AP8901 in the last case.

### 2.5 Sample size

The study is conducted using a 3 + 3 design, commonly used in phase I trials of anticancer drugs, to ensure subject safety. Three to six patients will be clustered at each dose level, with an anticipated planned enrollment of up to 18 patients. The trial targets EPHB4-expressing solid tumors, including Ewing sarcoma. The Bone and Soft-Tissue Tumor Registry estimates that there are 500–800 patients with primary bone sarcoma annually in Japan, including an estimated 10% (50 patients) with Ewing sarcoma. The planned enrollment period is 2 years, from October 2023 to September 2025, and the planned follow-up period is up to 1 year from the date of administration of AP8901 in the last patient.

### 2.6 Statistical analysis

DLT is calculated as the proportion at each dose level. The proportion and 95% confidence interval will be estimated for ORR and DCR. The results for subgroups will be compared using Fisher’s exact tests. PFS, OS, and DoR will be calculated by the Kaplan–Meier method and comparisons between subgroups will be made using the log-rank test and Cox proportional hazards model.

## 3 Discussion

**Figure 2:**
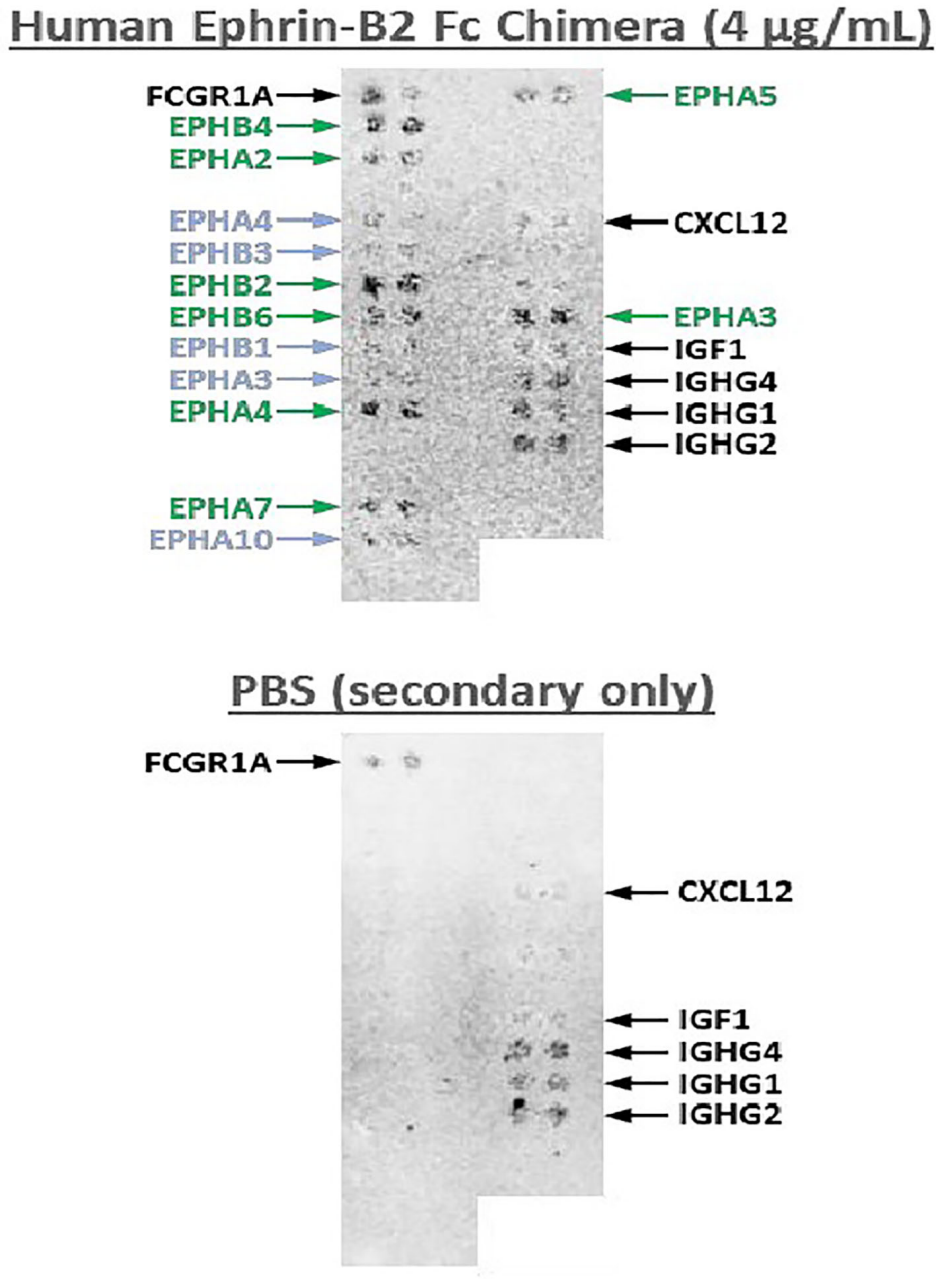
Cross-reactivity test of Human Ephrin-B2 Fc Chimera Protein with human cell surface proteins. A cross-activity test was conducted with 5868 human membrane and surface-anchored secreted proteins and Recombinant Human Ephrin-B2 Fc Chimera Protein (4 µg/mL). The results demonstrated that Recombinant Human Ephrin B2 Fc Chimera Protein binds specifically to EPHB4 and to the Eph family proteins EPHB2, EPHA2, EPHA3, EPHB4, EPHA5, EPHA7, and EPHB6, with very weak reactivity with EPHA5 and EPHA7.

This is a first-in human phase 1 study of EPHB4-CAR-T cell therapy. The advantage of AP8901 is that it is expected to prevent T cell exhaustion and maintain its anti-tumor effect. In a preclinical trial, EPHB4-CAR-T cells co-cultured with RH30 cells for 3 days inhibited tumor cell proliferation with no attenuation of cytotoxic activity (24). EPHB4-CAR-T cells also demonstrated activity in an animal model, with no serious adverse events (24). Regarding AP8901, potential cross-reactivity of EPHB4 CAR molecules with other ephrin family member proteins is a potential concern, given that the structure of ephrin proteins is highly conserved. We tested the cross-reactivity of Recombinant Human Ephrin-B2 Fc Chimera Protein with 5868 human membrane proteins and surface-anchored secreted proteins (**Figure 2**). The AP8901 Recombinant Human Ephrin B2 Fc Chimera Protein was suggested to bind to EPHB4, but also to the Eph family proteins EPHB2, EPHA2, EPHA3, and EPHB4, and very weak reactivity with EPHA5, EPHA7, and EPHB6 was also suggested. However, human and murine EPH family proteins are highly conserved, and the EPHB4 CAR protein can bind with murine EPH family proteins (**Figure 2**), thus enabling the assessment of off-tumor toxicity in an immunodeficient murine xenograft model. Indeed, a previous preclinical study found no off-tumor toxicities in an immunodeficient murine xenograft model treated with EPHB4 CAR-T cells (25). The potential on-target/off-target organ toxicities include the following: central nervous system disorder, eyes (inflammation of the cornea), cardiovascular injury, skin disorder, nasopharyngitis, mastitis, musculoskeletal and connective tissue inflammation, lymphadenitis, endometritis, nephritis. We plan to monitor these syndromes with clinical and laboratory findings during the observation period.

Additionally, we will monitor long-term survival as well as second malignancies as a potential adverse effect of CAR-T cell therapy (27). The current trial includes a supplemental 5-year long-term follow-up study and specimen collection and analysis at the time of second cancer occurrence.

In conclusion, this phase 1 study of AP8901 will provide new evidence for the application of this novel CAR-T cell therapy in patients with solid tumors, including Ewing sarcoma.
